# TP53 mitigates cisplatin resistance in non-small cell lung cancer by mediating the effects of resistant cell-derived exosome mir-424-5p

**DOI:** 10.1016/j.heliyon.2024.e26853

**Published:** 2024-02-22

**Authors:** Yan Deng, Hao Ding, Yanhua Zhang, Xudong Feng, Qing Ye, Rui Tian, Yuchuan Xu, Qingqing He, Qiaofen Fu, Rongqing Li

**Affiliations:** a Department of Radiotherapy, the First Affiliated Hospital of Kunming Medical University, No.295 Xichang Road, Kunming, Yunnan Province, 650032, China

**Keywords:** Non-small cell lung cancer, Cisplatin resistance, Exosome, Tumor protein p53, MicroRNA

## Abstract

**Background:**

Cisplatin (DDP) is the principal agent used for chemotherapy in patients with non-small cell lung cancer (NSCLC). Nevertheless, DDP resistance is an essential cause for a worse prognosis of patient. Therefore, this study proposes to discover features of miR-424-5p in DDP resistance of NSCLC.

**Method:**

After exogenous modulation of miR-424-5p expression, A549 cell activity was measured using CCK-8 and flow cytometry. A549/DDP and A549/DDP-associated subcutaneous tumor model were constructed to investigate the effect of miR-424-5p on DDP resistance in NSCLC *in vivo*. TargetScan and JASPAR databases predicted the potential molecular mechanism of miR-424-5p. A549-and A549/DDP-derived exosomes were isolated and characterized using a transmission electron microscope and nanoparticle tracking analysis.

**Result:**

Overexpression of miR-424-5p facilitated proliferation and DDP resistance in A549 cells, and knockdown of miR-424-5p did the opposite. Knockdown of miR-424-5p enhanced DDP restriction on tumor weight and volume. Moreover, SOCS5 and SOCS56 (SOCS5/6) were downstream targets of miR-424-5p. miR-424-5p down-regulated SOCS5/6 expression to activate JAK2/STAT3 and PI3K/AKT pathways. Notably, tumor protein p53 (TP53) is a transcription factor for the miR-424-5p host gene, as confirmed by the dual-luciferase reporter gene. Cellular and animal experiments indicated that TP53 limited the regulatory function of miR-424-5p on NSCLC growth, DDP resistance, and related molecules. Interestingly, miR-424-5p was markedly enriched in A549/DDP cell-derived exosomes than in A549 cell-derived exosomes, and TP53 down-regulated miR-424-5p expression in A549/DDP cell-derived exosomes.

**Conclusion:**

DDP-resistant cell-derived exosome miR-424-5p contributes to NSCLC growth and DDP resistance by targeting SOCS5 and SOCS6 to activate JAK2/STAT3 and PI3K/AKT pathways, which are blocked by TP53.

## 1 Introduction

Non-small cell lung cancer (NSCLC) is widespread worldwide and ranks first in cancer mortality [1,2]. Similarly, lung cancer ranks first in China in terms of cancer incidence and mortality and is still on the rise [3]. With the development of NSCLC treatment, immunotherapy and targeted therapy have been widely applied in the practice. Platinum-based chemotherapy remains one of the more effective treatments for patients with intermediate and advanced NSCLC, including cisplatin (DDP), carboplatin and oxaliplatin, etc. [4,5]. Nevertheless, patients with NSCLC treated with platinum have a worse outcome, with about 10% survival rate at 5 years including DDP, carboplatin, nedaplatin, and oxaliplatin [6,7]. In recent years, platinum resistance has become a leading cause of treatment failure and high fatality in NSCLC [4]. Therefore, it is important to explore the mechanism of platinum resistance and find the sensitization method.

MicroRNAs (miRNAs) are a set of short-sequence RNAs that do not encode proteins and consist of 21–25 nucleotides. Differential miRNA expression in tumors not only regulates tumor cell survival and metastasis, but also modulates chemosensitivity [8,9]. Currently, miRNAs regulate tumor phenotypes mainly by target-binding to the mRNA 3′-untranslated region (3′-UTR), thereby suppressing the expression of target genes [8,10]. Our previous screening of clinical samples revealed that miR-424 was highly expressed in NSCLC tissues with DDP-resistant [11]. Accordingly, miR-424 may be an important driver of DDP resistance in NSCLC. MiR-424 is a part of the miR-16 family, which is primarily responsible for regulating cell cycle, apoptosis, and other processes [12,13]. Previous study indicated that miR-424-5p was highly expressed in NSCLC, renal cancer, and pancreatic cancer, and lowly expressed in cervical, ovarian, and other reproductive system tumors [12,13]. Interestingly, miR-424 has different biology functions in different kinds of tumors. MiR-424 can act as an oncogene to promote tumor growth as well as as an anti-oncogene to inhibit tumor growth, which may be related to different in the tumor microenvironments and the binding to different target genes [12,14]. In NSCLC, studies of miR-424-5p have focused on its expression and prognosis, but its relationship with DDP resistance and the specific mechanism has not been elucidated yet.

In summary, this study intends to investigate miR-424-5p effects on NSCLC proliferation, cell cycle, apoptosis, and DDP resistance in cell and animal experiments, and to explore its potential molecular mechanisms. It aims to provide more references on the role of miR-424-5p in the malignant biological behavior and DDP resistance of NSCLC, and to offer new perspectives and therapeutic strategies for miR-424-5p as a target.

## 2 Materials and methods

### 2.1 Cell culture

A549 (Art. No.: CL-0016) and DDP-resistant cell A549/DDP (Art. No.: CL-0519) obtained from Procell (China). A549 and A549/DDP cells were cultured in Roswell Park Memorial Institute (RPMI)-1640 medium (11875119; Gibco, USA) and A549/DDP cell-specific medium (CM-0519, Procell) containing 10% fetal bovine serum (16140089; Gibco), respectively. When the cell density reached 80%–90%, digestion was performed using 0.25% trypsin (15090046; Gibco) for passaging.

### 2.2 Cell transfection and treatment

pCDNA3.1(+)-tumor protein p53 (TP53) and pGMLV-SC5 RNAi-TP53 plasmids, miR-424-5p mimic/inhibitor, as well as their negative controls were synthesized by Genomeditech (China). The targeting sequence of pGMLV-SC5 RNAi-NC was 5′-TTCTCCGAACGTGTCACGT-3'. The targeting sequences of pGMLV-SC5 RNAi-TP53 were 5′-GCTCAGATAGCGATGGTCTGG-3′, 5′-GCGTGTGGAGTATTTGGATGA-3′ and 5′-GGAAGACTCCAGTGGTAATCT-3'. A549 cells was randomized into Control, NC mimic, NC inhibitor, miR-424-5p mimic, miR-424-5p inhibitor, sh-NC, sh-TP53, pcDNA3.1-NC, and pcDNA3.1-TP53 groups. A549/DDP cells was randomly divided into Con, OV-NC, KD-NC, OV-miR-424-5p, KD-miR-424-5p, KD-TP53, and OV-TP53 groups. Referring to the Lipofectamine® 3000 Transfection Kit (L3000015; Invitrogen, USA) instructions, pCDNA3.1(+)-TP53, pGMLV -SC5 RNAi-TP53, miR-424-5p mimic/inhibitor and their negative controls were transfected. All groups of A549/DDP cells were transfected under IC_50_ DDP (HY-17394; MCE, USA) treatment. After 24 h of transfection, A549 and A549/DDP cells of each group were collected.

### 2.3 Dual-luciferase report gene assay

TargetScan was applied to predict downstream mRNAs of miR-424-5p, and the JASPAR was utilized to forecast transcription factors of miR-424-5p host gene (NC_000023.11). The downstream mRNAs were finally identified as SOCS5 and SOCS6, and the transcription factor as TP53. PGL3-CMV-LUC-MCS-SOCS5-WT/MUT, PGL3-CMV-LUC-MCS-SOCS6-WT/MUT, and PGL3-basic-has-miR-424-5p promoter (−2000to-1) WT/MUT vectors were synthesized by Genomeditech (China). PGL3-CMV-LUC-MCS-SOCS5-WT/MUT and PGL3-CMV-LUC-MCS-SOCS6-WT/MUT vectors were co-transfected with miR-NC and miR-24-3p mimic into 293T cells, respectively. PGL3-basic-has-miR-424-5p promoter (−2000to-1) WT/MUT vector was co-transfected with pCDNA3.1(+)-NC and pCDNA3.1(+)-TP53 vectors into 293T cells, respectively. After transfection for 48 h, the absorbance of each group of cells was measured using a GloMax® Luminescence Detector (E6521; E6521).

### 2.4 Isolation and identification of exosomes

A549 and A549/DDP cells were exchanged for serum-free RPMI-1640 medium, and cultured for 24 h. The supernatants were subjected to centrifugation, to obtain the exosome precipitates, which were named A549-exo and A549/DDP-exo, respectively. Transmission electron microscopy (TEM) and nanoparticle tracking analysis (NTA) were performed to observe the morphology and size of A549-exo and A549/DDP-exo. For TEM, A549-exo and A549/DDP-exo were added to a carrier mesh with a carbon-coated Formvar film. Exosomes in the carrier mesh were fixed with 2.5% glutaraldehyde for 5 min, and stained with 40 g/L uranium acetate and 10 g/L methylcellulose for 10 min and 5 min, respectively. After the carrier mesh was dried, an HT7800 transmission electron microscope (HITACHI, Japan) was used for image collection of A549-exo and A549/DDP-exo. For NTA, A549-exo and A549/DDP-exo solutions were observed using ZetaView® MONO (Particle-Metrix; Germany), and were analyzed for exosome concentration and diameter.

### 2.5 Western blotting assay

The total protein of A549 cells, A549/DDP cells, A549-exo, and A549/DDP-exo were extracted using RIPA lysate (89901; Thermo Scientific, USA) and Total exosomal RNA& Protein isolation kit (4478545; Invitrogen), and the total protein concentration of each sample was detected. An equal amount of total protein was taken to perform electrophoretic separation using sodium dodecyl sulfate-polyacrylamide gel electrophoresis (SDS-PAGE). Subsequently, proteins in SDS-PAGE were transferred to polyvinylidene fluoride (PVDF) membranes using the wet transfer method. PVDF membranes were closed with 5% bovine serum protein (ST023-50 g; Beyotime, China) for 1 h. PVDF membrane was then added with primary and secondary antibodies of target proteins. The antibody information is shown in Supplementary Table 1. PVDF membranes were color developed using Novex™ ECL Kit (WP20005; Invitrogen), and images were collected using an Axygen® Gel Imaging System (GD-1000; Corning, USA).

#### 2.5.1 RT-qPCR assay

Total RNA from A549 cells, A549/DDP cells, A549-exo, and A549/DDP-exo was extracted using Trizol™ reagent (15596026; Invitrogen) and Total exosomal RNA & Protein isolation kit, and the concentration of total RNA in each sample was determined using a NanoDrop-1000 ultra-micro spectrophotometer (Thermo Fisher). Reverse transcription and amplification of total RNA were performed to detect miR-424-5p expression, using Bulge-Loop miRNA qRT-PCR Starter Kit (C10211-1; Ribobio, China). TP53, SOCS5, and SOCS6 were detected using FastKing RT Kit (KR116; Tiangen, Germany) to perform reverse transcription, and Taq Pro Universal SYBR qPCR Master Mix Kit (Q712-03; Vazyme, China) to perform amplification. Primer sequences for U6, GAPDH, miR-424-5p, SOCS5, SOCS6, and TP53 are shown in Supplemental Table 2.

### 2.6 Animal and subcutaneous tumor model

BALB/c-nu mice (18–22 g) were obtained from Hunan Silaikejingda Co., LTD. All BALB/c-nu mice had free access to food and water, and were adaptively fed for 1 week for subsequent experiments. The feeding environment was an SPF environment with 23 ± 3 °C for temperature, 60 ± 5 % for humidity, and day/night alternation of 12/12 h. The ethical committee of the First Affiliated Hospital of Kunming Medical University authorized this study (Appl. No.: SL20220305). The study adhered strictly to the ARRIVE guidelines and was designed to minimize the number and pain suffered by the animal using anesthetics and analgesics, as well as the setting of endpoints for the experiments.

All BALB/c-nu mice were randomly divided into PBS, DDP, miR-424-5p, OV-TP53, and OV-TP53+OV-miR-424-5p groups, with six in each group. According to the grouping information, mice were injected subcutaneously on the dorsal side with transfected A549/DDP cells at 5 × 10^5^ cells per mice. After 7 d, mice in the DDP, OV-miR-424-5p, OV-TP53, and OV-TP53+OV-miR-424-5p groups were injected intraperitoneally with 5 mg/kg of DDP, with three times a week for two weeks. When neoplasm appeared subcutaneously in mice, the measurement of tumor volume was started and measured every 3 d. On 27 d, all mice were injected with an overdose of pentobarbital sodium (150 mg/kg) to perform euthanasia, and the tumors were collected for subsequent experiments.

### 2.7 Immunohistochemical (IHC) staining

Briefly, tumor tissue was fixed using 4% paraformaldehyde. Tumor tissue was prepared as 5 μm sections and deparaffinized using xylene. Tumor sections were subjected to antigenic repair using citrate buffer, and closed with 5% sheep serum. Subsequently, tumor sections were incubated with primary and secondary antibodies, respectively. The antibody information is shown in Supplementary Table 1. DAB substrate kit (ab64238; Abcam, USA) was used to develop the color of the tumor sections, and the section images were observed and collected using a BX53 microscope (Olympus, Tokyo, Japan).

### 2.8 Statistical analysis

All experiments were repeated at least three times, and the data were statistically analyzed using GraphPad Prism V.9.3.0 (GraphPad, CA, USA). Data that conformed to normal distribution and homogeneity of variance was analyzed using Student's t-test or one-way ANOVA. On the contrary, the non-parametric test is used. *P* < 0.05 indicates statistical significance.

## 3 Results

### 3.1 MiR-424-5p facilitates NSCLC growth and DDP resistance

**Fig. 1:**
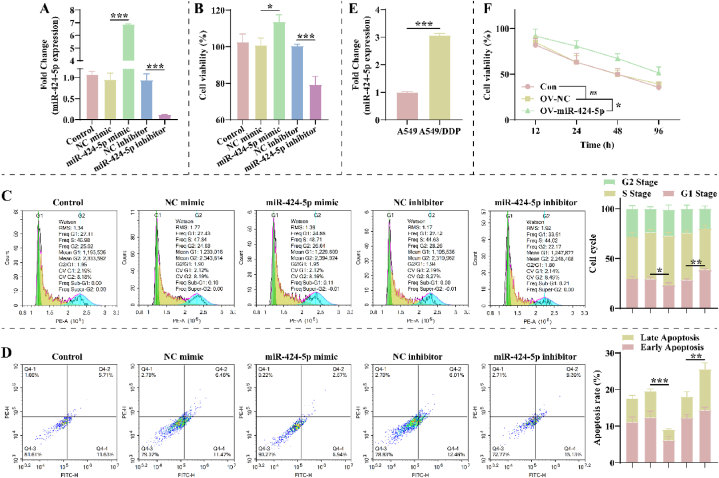
**miR-424-5p facilitates growth and cisplatin (DDP) resistance of A549 cells. A**: RT-qPCR assay was performed to detect the expression level of miR-424-5p in A549 cells after transfection with miR-424-5p mimic or inhibitor (n = 3). **B**: Cell counting kit-8 (CCK-8) assay was used to detect the proliferation of A549 cells in each group at 48 h (n = 3). **C**: After propidium iodide (PI) staining, the cell cycle of A549 cells in each group was detected by flow cytometry (n = 3). **D**: Annexin V-FITC/PI kit was utilized to measure the effect of miR-424-5p on apoptosis in A549 cells (n = 3). **E**: Differential expression of miR-424-5p in A549 and DDP-resistant cells (A549/DDP) (n = 3). **F**: Effect of over-expression of miR-424-5p on the proliferation of A549/DDP cells after IC_50_ DDP treatment was detected using CCK-8 (n = 3). The NC mimic, miR-424-5p mimic, NC inhibitor, and miR-424-5p inhibitor groups were transfected with NC mimic, miR-424-5p mimic, NC inhibitor, and miR-424-5p inhibitor for **A549 cells**, respectively. The OV-NC and OV-miR-424-5p groups were transfected with NC mimic and miR-424-5p mimic for **A549/DDP cells**, respectively. Control and Con groups were **A549** and **A549/DDP cells** without treatment.

To explore miR-424-5p function on the malignant phenotype of NSCLC, we exogenously regulated miR-424-5p expression in A549 cells. As illustrated in Fig. 1A, transfection of miR-424-5p mimic markedly increased its expression in A549 cells, and the opposite result was obtained after transfection of miR-424-5p inhibitor. Cell counting kit-8 (CCK-8) assay revealed that miR-424-5p mimic markedly enhanced A549 cell viability, and miR-424-5p inhibitor did the opposite (Fig. 1B). As expected, the G1-phase of A549 cells was lower markedly in the miR-424-5p mimic group and increased markedly in the miR-424-5p mimic group compared to the negative control group (Fig. 1C). Moreover, over-expressing miR-424-5p significantly reduced apoptosis in A549 cells, and the knockdown of miR-424-5p reversed it (Fig. 1D). Further, we examined the effect of miR-424-5p on NSCLC DDP resistance. Interestingly, miR-424-5p was over-expressed in A549 DDP-resistant cells (A549/DDP cells) (Fig. 1E). We treated with IC_50_ DDP in A549/DDP cells exogenously regulating miR-424-5p. CCK-8 demonstrated that over-expressing miR-424-5p increased the cell viability of DDP-treated A549/DDP cells (Fig. 1F).

**Fig. 2:**
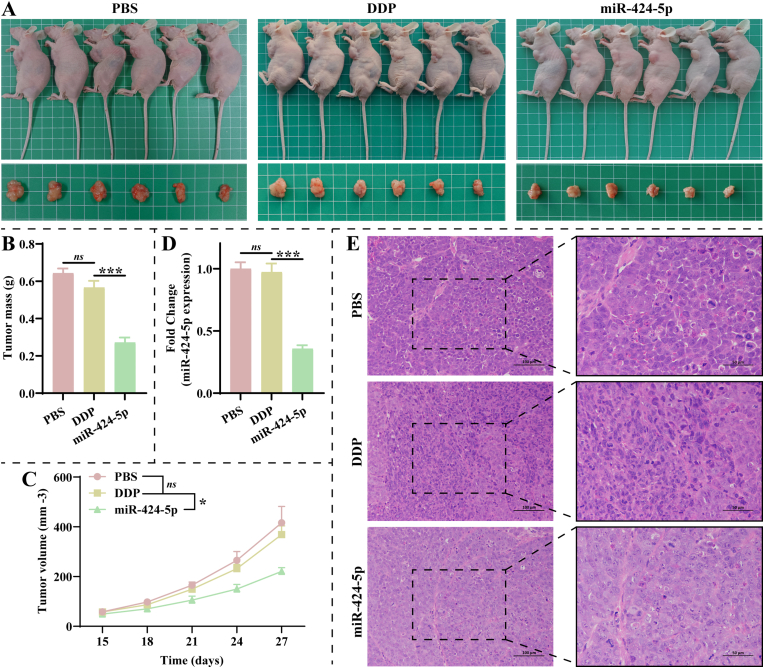
Knockdown of miR-424-5p alleviates growth and DDP resistance in non-small cell lung cancer (NSCLC) *in viv*o. **A**: Images of tumor tissue *in vivo* and *ex vivo* (n = 6). **B**: Tumor mass (g) of mice in each group (n = 6). **C**: Growth curves of tumor volume (mm^3^) in each group of mice from 15 d (n = 6). **D**: Differences in miR-424-5p expression in tumor tissues were detected by RT-qPCR (n = 3). **E**: Hematoxylin-eosin (HE) staining was performed to observe the effect of miR-424-5p on tumor histopathology (n = 3). Scale bar: 100 or 50 μm. Mice in the PBS and DDP groups were subcutaneously injected with untreated A549/DDP cells and treated with PBS and 5 mg/kg of DDP, respectively. Mice in the miR-424-5p group were subcutaneously injected with A549/DDP cells transfected with miR-424-5p inhibitor and treated with 5 mg/kg of DDP.

We further constructed a subcutaneous tumor model of A549/DDP cells to research the miR-424-5p effect on DDP resistance in NSCLC *in vivo*. Fig. 2A displays *in vivo* and *ex vivo* images of the tumor. There were no significant changes in tumor mass and volume between the PBS and DDP groups (Fig. 2B–C). Notably, tumor mass and volume were significantly lower in the miR-424-5p group than in the DDP group (Fig. 2B–C). As expected, miR-424-5p was reduced markedly in tumors of the miR-424-5p group (Fig. 2D). As shown in Fig. 2E, the PBS group exhibited active growth of tumor cells, with large and deeply stained nuclei, close cell arrangement, and frequent phenomenon of nuclear division. The DDP group displayed necrosis of tumor cells, chromatin condensation, less phenomenon of nuclear division, and nuclear fragmentation, with inflammatory cell infiltration. The miR-424-5p group had the least pathologic symptoms.

### 3.2 MiR-424-5p activates PI3K/AKT and JAK2/STAT3 pathways via regulating SOCS5/6

**Fig. 3:**
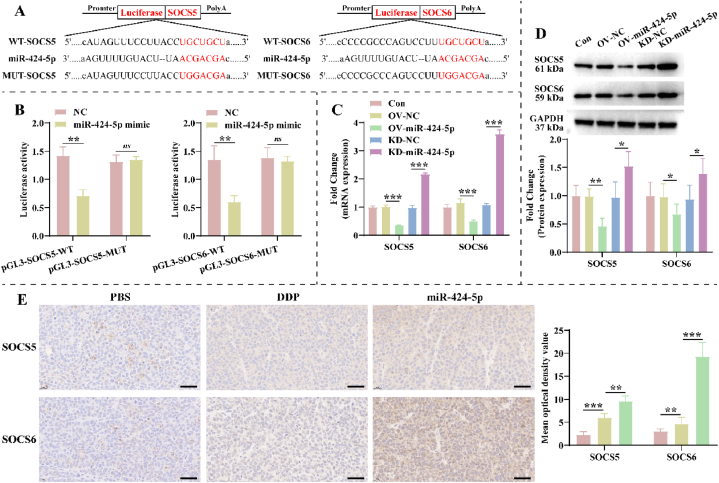
**SOCS5 and SOCS6 are target genes of miR-424-5p. A**: Binding sequences of miR-424-5p to SOCS5 and SOCS6 were predicted from the TargetScan database. **B**: Dual-luciferase reporter gene assay was performed to validate the targeted relationship of miR-424-5p with SOCS5 and SOCS6 (n = 3). **C-D**: (C) RT-qPCR and (D) western blotting were utilized to assess the effect of miR-424-5p on SOCS5 and SOCS6 expression in A549/DDP cells (n = 3). The original gel blot is shown in Fig. 3D of the Supplementary file. **E**: Mean optical density values of SOCS5 and SOCS6 were analyzed by immunohistochemical (IHC) staining in tumor tissues of PBS, DDP, and miR-424-5p groups (n = 3). Scale bar: 50 μm. The NC and miR-424-5p mimic groups were transfected with the NC mimic and miR-424-5p mimic for **293T cells**, respectively. The OV-NC, OV-miR-424-5p, KD-NC, and KD-miR-424-5p groups were transfected with NC mimic, miR-424-5p mimic, NC inhibitor, and miR-424-5p inhibitor for **A549/DDP cells**, respectively. The information of PBS, DDP, and miR-424-5p groups is described in Figure Legend 2.

MiRNAs regulate the expression of downstream mRNA through an RNA-induced silencing complex, which is the main pathway for their biological functions. Therefore, we predicted the potential miR-424-5p’ targets using the TargetScan database. As exhibited in Fig. 3A, the seed sequence of miR-424-5p was complementary to the 3′ UTRs of SOCS5 and SOCS6, which suggested that SOCS5 and SOCS6 might be downstream of miR-424-5p. To explore their relationship, we mutated the binding sequences of the 3′ UTRs of SOCS5 and SOCS6. Compared with the NC group, over-expressing miR-424-5p significantly reduced luciferase activity of wild-type SOCS5 and SOCS6 in 293T cells, without effects on mutant SOCS5 and SOCS6 (Fig. 3B). Moreover, SOCS5 and SOCS6 expression were lower markedly in the OV-miR-424-5p group than in the OV-NC group, and vice versa in the KD-miR-424-5p group (Fig. 3C–D). Notably, DDP treatment significantly increased SOCS5 and SOCS6 expression in tumor tissues, and this phenomenon was further enhanced after the knockdown of miR-424-5p (Fig. 3E). This suggests that miR-424-5p targets and inhibits the expression of SOCS5 and SOCS6 in NSCLC *in vitro* and *in vivo*.

**Fig. 4:**
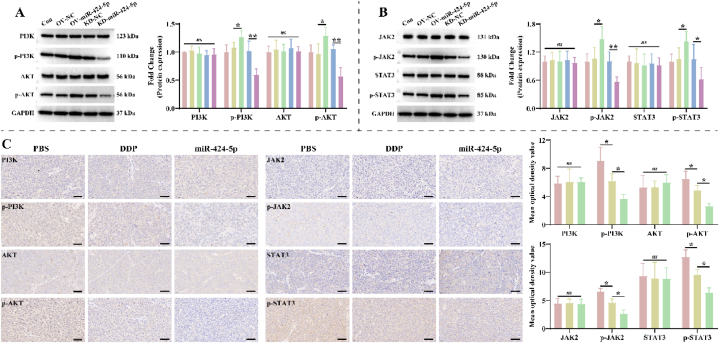
**miR-424-5p activates PI3K/AKT and JAK2/STAT3 pathways in A549/DDP cells. A-B**: Effects of miR-424-5p on the activity of (A) PI3K/AKT and (B) JAK2/STAT3 pathways in A549/DDP cells were detected by Western blotting (n = 3). The original gel blot is shown in Fig. 4A–B of the Supplementary file. **C**: IHC representative pictures of tumor tissues from PBS, DDP, and miR-424-5p groups regarding PI3K/AKT and JAK2/STAT3 pathways (n = 3). Scale bar: 50 μm. The information of Con, OV-NC, OV-miR-424-5p, KD-NC, KD-miR-424-5p, PBS, DDP, and miR-424-5p groups is as described in Figure Legend 3.

Notably, the JAK2/STAT3 and PI3K/AKT pathways have been demonstrated to be regulated by SOCS5 and SOCS6 in a variety of malignancies, and they are regulated DDP resistance in NSCLC. Therefore, we further explored the effects of miR-424-5p on the activity of JAK2/STAT3 and PI3K/AKT pathways. Over-expression of miR-424-5p up-regulated the phosphorylation levels of PI3K, AKT, JAK2, and STAT3 in A549/DDP cells, but did not affect total protein expression (Fig. 4A–B). The opposite result was obtained by knocking down miR-424-5p (Fig. 4A–B). Tissue staining indicated that the mean optical density values of p-PI3K, *p*-AKT, *p*-JAK2, and p-STAT3 were significantly lower in the DDP group compared to the PBS group, and more pronounced in the miR-424-5p group (Fig. 4C). This suggests that miR-424-5p suppress the expression of SOCS5 and SOCS6 to activate JAK2/STAT3 and PI3K/AKT pathways, which is responsible for the promotion of NSCLC growth and DDP resistance.

### 3.3 TP53 mediates miR-424-5p expression in NSCLC

**Fig. 5:**
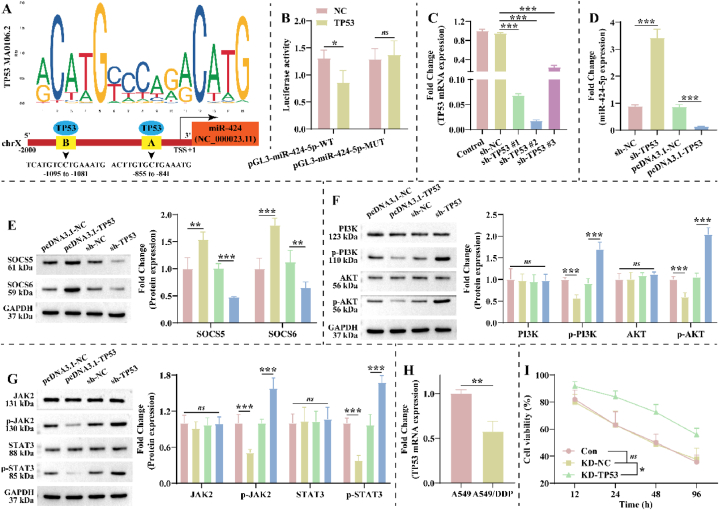
**TP53 regulates miR-424-5p expression in A549/DDP cells. A**: JASPAR (http://jaspar.binf.ku.dk/) predicted transcription factors regulating the miR-424 host gene (NC_000023.11). **B**: The targeted relationship of TP53 to the miR-424 host gene (NC_000023.11) was validated in 293T cells using dual-luciferase reporter gene assay (n = 3). **C**: Transfection efficiency of sh-TP53 in A549 cells was checked by RT-qPCR (n = 3). **D**: Effect of TP53 on miR-424-5p expression in A549 cells (n = 3). **E-G**: Western blotting was performed to assess the effect of TP53 on (E) SOCS5 and SOCS6 expression, and the activity of (F) PI3K/AKT and (G) JAK2/STAT3 pathways (n = 3). The original gel blot is shown in Fig. 5E–G of the Supplementary file. **H**: Differences in miR-424-5p expression were analyzed by RT-qPCR in A549 and A549/DDP cells (n = 3). **I**: The effect of the knockdown of TP53 on the proliferation of A549/DDP cells was detected by CCK-8 under DDP treatment (n = 3). The NC and TP53 groups were transfected with pCDNA3.1(+)-NC and pCDNA3.1(+)-TP53 for **293T cells**, respectively. The sh-NC, sh-TP53, pcDNA3.1-NC and pcDNA3.1-TP53 groups. were transfected with pGMLV-SC5 RNAi-NC, pGMLV-SC5 RNAi-TP53, pCDNA3.1(+)-NC, and pCDNA3.1(+)-TP53 for **A549 cells**, respectively. The KD-NC and KD-TP53 groups were transfected with pGMLV-SC5 RNAi-NC, and pGMLV-SC5 RNAi-TP53 for **A549/DDP cells**, respectively.

To explore a more complete molecular mechanism, we predicted transcription factors of the miR-424-5p host gene using JASPAR (http://jaspar.binf.ku.dk/). The host gene for miR-424-5p is NC_000023.11, located at Xq26.3 (134546614–134546711). We selected 2000 bp upstream of the transcription start site (TSS) to be set as the promoter region of NC_000023.1. As displayed in Fig. 5A and Supplementary Table 3, the motif of TP53 has two binding sites to the promoter region of NC_000023.11, which are TCATGTCCTGAAATG and ACTTGTGCTGAAATG. Further, we mutated the sequences of two sites in NC_000023.11 and validated the targeting relationship. The results revealed that over-expressing TP53 significantly decreased the luciferase activity of the pGL3-miR-424-5p-WT vector in 293T cells, without effect on pGL3-miR-424-5p-MUT (Fig. 5B). Further, we explored the effect of TP53 on the expression of miR-424-5p, SOCS5 and SOCS6, and the activity of JAK2/STAT3 and PI3K/AKT pathways in A549 cells. Transfection of shRNA significantly reduced TP53 expression in A549 cells, and the effect was most pronounced for sh-TP53 #2 (Fig. 5C). We selected sh-TP53 #2 for subsequent experiments. TP53 could negatively regulate miR-424-5p expression in A549 cells (Fig. 5D). In addition, the knockdown of TP53 down-regulated SOCS5 and SOCS6 expression and up-regulated p-PI3K, *p*-AKT, *p*-JAK2, and p-STAT3 expression in A549 cells (Fig. 5E–G). Opposite results were obtained in A549 cells over-expressing TP53 (Fig. 5E–G). Notably, we found that the expression level of TP53 was significantly lower in A549/DDP cells than in A549 cells (Fig. 5H). CCK-8 demonstrated that knockdown of TP53 enhanced DDP resistance in A549/DDP cells (Fig. 5I). Combined with previous findings, this suggests that TP53 is upstream of miR-424-5p and up-regulates SOCS5 and SOCS6 expression to inhibit JAK2/STAT3 and PI3K/AKT pathways activity, which alleviates NSCLC growth and DDP resistance.

### 3.4 TP53 mitigates NSCLC growth and DDP resistance through miR-424-5p *in vivo*

**Fig. 6:**
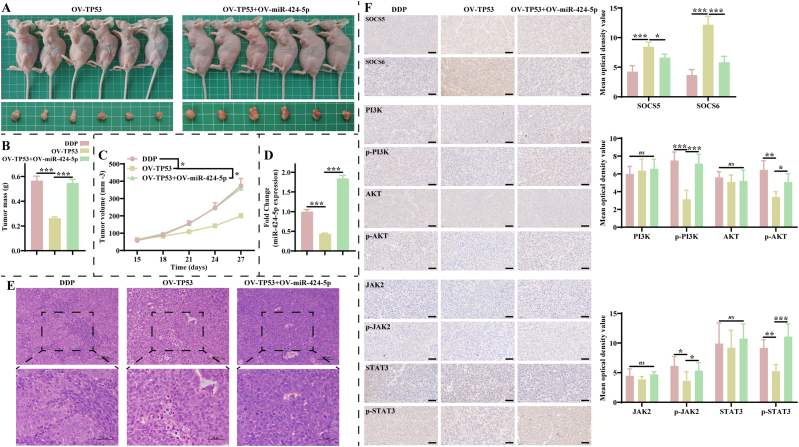
**TP53 mitigates NSCLC growth and DDP resistance through miR-424-5p *in vivo*. A**: Representative images of mice and tumors in the OV-TP53 and OV-TP53+OV-miR-424-5p groups (n = 6). **B–C**: Overexpression of TP53 enhanced the inhibitory effect of DDP on (B) tumor mass and (C) volume (n = 6). **D**: RT-qPCR exhibited that over-expression of TP53 suppressed miR-424-5p expression in tumor tissues (n = 3). **E**: HE representative images of tumor tissues from DDP, OV-TP53, and OV-TP53+OV-miR-424-5p groups (n = 3). Scale bar: 100 or 50 μm. **F**: IHC representative images of PI3K/AKT and JAK2/STAT3 pathways in tumor tissues of each group (n = 3). Scale bar: 50 μm. Mice in the DDP, OV-TP53, and OV-TP53+OV-miR-424-5p groups were subcutaneously injected with untreated, pCDNA3.1(+)-TP53-transfected and pCDNA3.1(+)-TP53- and miR-424-5p mimic-transfected A549/DDP cells, and were treated with 5 mg/kg DDP, respectively.

Further, we validated the function of TP53 in the subcutaneous tumor model. Images of mice and tumors in the OV-TP53 and OV-TP53+OV-miR-424-5p groups are shown in Fig. 6A. Statistics revealed that the mass and volume of the tumor were lower significantly in the OV-TP53 group compared to the DDP group, and this phenomenon was restored in the OV-TP53+OV-miR-424-5p group (Fig. 6B–C). Moreover, over-expression of TP53 markedly decreased miR-424-5p expression in the tumor, while simultaneous over-expression of TP53 and miR-424-5p restored the expression of miR-424-5p (Fig. 6D). Hematoxylin-eosin (HE) staining exhibited that, compared with the DDP group, the tumor tissue appeared to have disorganized cell arrangement, with different degrees of necrosis and infiltration of inflammatory cells in the OV-TP53 group, and this pathological change was reversed in the OV-TP53+OV-miR-424-5p group (Fig. 6E). As expected, over-expression of TP53 resulted in a significant decrease of p-PI3K, *p*-AKT, *p*-JAK2, and p-STAT3 expression, without effect on the expression of their total proteins (Fig. 6F). Similarly, simultaneous over-expression of TP53 and miR-424-5p reversed the expression of four phosphorylated proteins (Fig. 6F). The above results suggest that the carcinogenic effects of miR-424-5p *in vivo* are limited by TP53.

### 3.5 Exosome is a functional medium for miR-424-5p

**Fig. 7:**
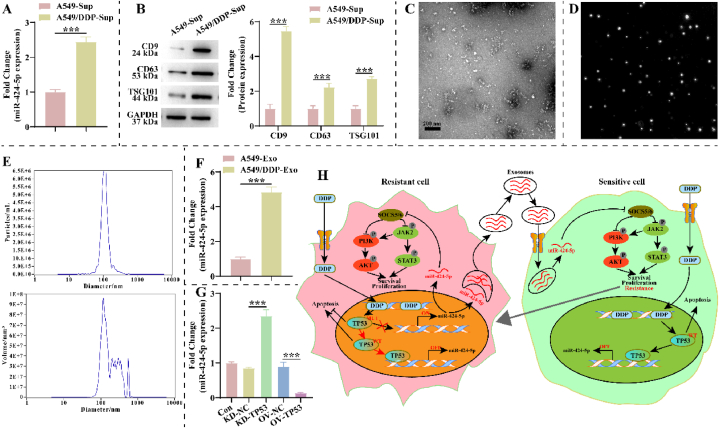
**Exosome is a functional medium for miR-424-5p. A**: Differences in miR-424-5p expression in supernatants of A549 and A549/DDP cells were detected by RT-qPCR (n = 3). **B**: Western blotting was performed to detect differences in the expression of exosomal membrane protein markers (CD9 and CD63) and intramembrane protein marker (TSG101) in the supernatants of A549 and A549/DDP cells (n = 3). The original gel blot is shown in Fig. 7B of the Supplementary file. **C**: Transmission electron microscope (TEM) representative images of isolated exosomes (n = 3). Scale bar: 200 nm. **D**: Nanoparticle tracking analysis (NTA) representative pictures of isolated exosomes (n = 3). **E**: Diameter distribution of isolated exosomes, and the corresponding particles and volumes of vesicles with different diameters (n = 3). **F**: Expression of miR-424-5p was significantly increased in A549/DDP cell-derived exosomes (n = 3). **G**: The effect of TP53 on miR-424-5p expression was analyzed by RT-qPCR in A549/DDP cell-derived exosomes (n = 3). **H**: Diagram of the molecular mechanism of this study. The A549-Sup and A549/DDP-Sup groups are A549-and A549/DDP-derived supernatants. The A549-Exo and A549/DDP-Exo groups are A549-and A549/DDP-derived exosomes.

Exosomes are important mediators of cellular communication, and miRNAs are one of the major substances in exosomes. This research probed whether exosomes are involved in regulating NSCLC growth and DDP resistance with miR-424-5p. We first examined miR-424-5p expression in the supernatants of A549 and A549/DDP cells. miR-424-5p expression was higher significantly in the supernatants of A549/DDP cells (Fig. 7A). Interestingly, we also found that the expression of both exosome membrane protein (CD9 and CD63) and intramembrane protein marker (TSG101) was increased markedly in the supernatants of A549/DDP cells compared to the supernatants of A549 cells (Fig. 7B). This suggests that A549/DDP cells secrete more exosomes, and exosomes may contribute to miR-424-5p-mediated DDP resistance. Therefore, we isolated A549 cell- and A549/DDP cell-derived exosomes. As displayed in Fig. 7C–D, the isolated vesicles were round or cup-shaped and had diameters around 60–160 nm (Fig. 7E). Consistently with cell supernatants, miR-424-5p expression was increased significantly in A549/DDP cell-derived exosomes compared to A549/DDP cell-derived exosomes (Fig. 7F). Remarkably, after exogenous regulation of TP53 in A549/DDP cells, miR-424-5p expression was altered in the exosomes from which it originated (Fig. 7G). This suggests that the A549/DDP cell-derived exosome miR-424-5p leads to the development of DDP resistance, which is restricted by TP53 (Fig. 7H).

## 4 Discussion

DDP inhibits cell division and proliferation by binding to DNA, thereby alleviating tumor progression. However, some patients with NSCLC may develop resistance in tumor cells after receiving DDP chemotherapy. This limits the efficacy of DDP, and increases the risk of recurrence and metastasis. Therefore, exploring the molecular mechanisms of DDP resistance in NSCLC can help develop new therapeutic strategies to overcome DDP resistance. In the present study, we uncovered that miR-424-5p is a booster of NSCLC growth and DDP resistance, and DDP-resistant cell-derived exosomes are mediators of the role of miR-424-5p. Notably, TP53 is a transcription factor for the miR-424-5p host gene, and the role of miR-424-5p in NSCLC growth and DDP resistance was restricted by TP53.

In fact, part of the miR-424-5p function in NSCLC has been demonstrated in previous studies. Li et al. [15] demonstrated that miR-424-5p acted as an oncogenic factor for promoting NSCLC proliferation and inhibiting apoptosis by targeting and suppressing the expression of PLSCR4. This is consistent with the findings of this study. In contrast, Drasin [16] and Wu [17] et al. suggested that miR-424-5p acted as a tumor suppressor, and alleviated proliferation, invasion, migration, and radio-resistance of NSCLC. It is noteworthy that both studies have been retracted. This suggests that their results are controversial. Compared to these studies, we further complement the role of miR-424-5p in the DDP resistance of NSCLC, and revealed that it can regulate SOCS5, SOCS6, and JAK2/STAT3 and PI3K/AKT pathways. Interestingly, the role of SOCS5, SOCS6, JAK2/STAT3, and PI3K/AKT pathways in DDP resistance of tumor cells has been well documented. Numerous researches have indicated that SOCS5 and SOCS6 act as cancer suppressors in NSCL [[18], [19], [20], [21]]. Furthermore, Sun et al. [22] demonstrated that over-expression of SOCS6 inhibited tumor cell growth and metastasis, and increased the DDP sensitivity of tumor cells. Notably, SOCS5 and SOCS6 have been well demonstrated to be inhibitors of activation of the JAK2/STAT3 and PI3K/AKT pathways [[23], [24], [25], [26]]. It is well known that the JAK2/STAT3 and PI3K/AKT pathways are important pathways for inducing DDP resistance in NSCLC [[27], [28], [29], [30]]. In the present study, we found that SOCS5 and SOCS6 are downstream targets of miR-424-5p, and miR-424-5p activated JAK2/STAT3 and PI3K/AKT pathways in NSCLC by targeting down-regulation of SOCS5 and SOCS6. Combined with previous studies, we believe that the promotion of miR-424-5p on NSCLC growth and DDP resistance is achieved by modulating SOCS5, SOCS6 JAK2/STAT3, and PI3K/AKT pathways.

The classical anti-cancer mechanism of DDP suggests that DDP binds to DNA to form DDP-DNA complexes resulting in DNA damage, and inducing cell death mediated by transcription factors, such as TP53 [31,32]. Therefore, stabilization and activation of wild-type TP53 is one of the critical factors in DDP-induced cell death. A large number of clinical findings have revealed that 36% of patients with NSCLC have primary TP53 mutations [33] and that patients with wild-type TP53 are more likely to benefit from DDP therapy than those with mutant TP53 [34,35]. Notably, we previously found that miR-424 expression showed a negative correlation with wild-type TP53 expression in NSCLC [11]. In the present study, we first proved that transcription factor TP53 binds to the promoter region of the host gene to inhibit miR-424-5p expression and affect the expression of SOCS5 and SOCS6 and the activity of JAK2/STAT3 and PI3K/AKT pathways. Combined with previous studies, our findings further explain the miR-424-5p regulation of NSCLC growth and DDP resistance. Exosomes are membrane vesicles about 30–160 nm in size that are actively secreted by many types of cells [36]. Exosomes contain proteins, DNA, mRNA, and miRNA, and can transmit these substances as signals to target cells [37]. Numerous studies have demonstrated that exosomes serve as a transporter for miRNA to deliver miRNAs to target cells, thereby modulating the sensitivity of target cells to chemotherapy in many cancer types [37,38]. For instance, Ran et al. [38] demonstrated that the cancer-associated fibroblast-derived exosome miR-3173-5p contributes to gemcitabine resistance in pancreatic cancer by mediating ACSL4-regulated ferroptosis. Interestingly, we confirmed that miR-424-5p was enriched in supernatants and exosomes of NSCLC cells and was more pronounced in DDP-resistant A549 cells. This suggests that exosomes are one of the mechanisms by which miR-424-5p exerts its DDP-resistant effects in NSCLC cells. Furthermore, exogenous regulation of TP53 in A549/DDP cells resulted in the altered expression of miR-424-5p in exosomes. Combined with other findings from this study, we believe that the DDP-resistant NSCLC cell-derived exosome miR-424-5p contributes to DDP resistance in normal NSCLC cells, and this process is restricted with TP53. This finding further emphasizes that miR-424-5p may act as a therapeutic target for DDP resistance in NSCLC.

In conclusion, this study found that TP53 regulates miR-424-5p expression and miR-424-5p is transported by exosomes. DDP-resistant cell-derived exosome miR-424-5p induces growth and DDP resistance of recipient cells by targeting and inhibiting SOCS5/6 expression to activate PI3K/AKT and JAK/STAT3 pathways. Exosome miR-424-5p may serve as a valuable biomarker for dynamic monitoring in patients with NSCLC and may potentially act as a key mediator in reversing DDP resistance in NSCLC.

## Data availability statement

Data will be made available on request.

## Funding

1. The Joint Special Funds for the Department of Science and Technology of Yunnan Province-Kunming Medical University (grant No. 202001AY070001-006).
2. The Joint Special Funds for the Department of Science and Technology of Yunnan Province-Kunming Medical University (grant No. 202001AY070001-141).
3. Precision Radiotherapy Science and Technology Innovation Team of Yunnan Universities (grant No. K12322113).
4. The Applied Basic Science Research Foundation of Yunnan Province (grant No. 202201AT070294).

## CRediT authorship contribution statement

**Yan Deng:** Writing – original draft, Visualization, Methodology, Investigation, Formal analysis, Conceptualization. **Hao Ding:** Writing – original draft, Visualization, Methodology, Investigation, Formal analysis, Conceptualization. **Yanhua Zhang:** Writing – review & editing, Methodology, Investigation. **Xudong Feng:** Writing – review & editing, Methodology, Investigation. **Qing Ye:** Writing – review & editing, Methodology, Investigation. **Rui Tian:** Writing – review & editing, Methodology, Investigation. **Yuchuan Xu:** Writing – review & editing, Visualization, Formal analysis. **Qingqing He:** Writing – review & editing, Visualization, Formal analysis. **Qiaofen Fu:** Writing – review & editing, Visualization, Formal analysis, Data curation. **Rongqing Li:** Writing – review & editing, Resources, Funding acquisition.

## Declaration of competing interest

The authors declare that they have no known competing financial interests or personal relationships that could have appeared to influence the work reported in this paper.
