# Supplementary material for: TP53 mitigates cisplatin resistance in non-small cell lung cancer by mediating the effects of resistant cell-derived exosome mir-424-5p
Source: Heliyon. 2024 Feb 22;10(5):e26853. doi: 10.1016/j.heliyon.2024.e26853 (PMC10909722; doi:10.1016/j.heliyon.2024.e26853)
Supplement: Multimedia component 1 [file mmc1.docx]

**Supplementary table 1. Antibody information for target protein.**

| Antibody | Producers | Art.No. | Dilution ratio/Concentration | |
| --- | --- | --- | --- | --- |
|  |  |  | WB | IHC |
| **Primary antibody** | | | | |
| TP53 | Bioss | bs-2090R | 1:500 | 1:200 |
| SOCS5 | Bioss | bs-13664R | 1: 1000 | 1:500 |
| SOCS6 | Bioss | bs-10685R | 1: 1000 | 1:5500 |
| PI3K | Bioss | bs-10657R | 1:1000 | 1:200 |
| p-PI3K | Bioss | bs-6417R | 1:1000 | 1:200 |
| AKT | Bioss | bs-6951R | 1:2000 | 1:500 |
| p-AKT | Bioss | bs-5193R | 1:2000 | 1:500 |
| JAK2 | Bioss | bs-23003R | 1:1000 | 1:300 |
| p-JAK2 | Abcam | ab32101 | 1:1000 | 1:500 |
| STAT3 | Bioss | bsm-33223M | 1:1000 | 1:200 |
| p-STAT3 | Bioss | bs-22386R | 1:1000 | 1:200 |
| GAPDH | Affinity | AF7021 | 1:5000 | 1:50 |
| **Second antibody** | | | | |
| Anti-rabbit IgG, HRP-linked Antibody | CST | 7074 | 1:2000 | 1:1000 |
| Anti-mouse IgG, HRP-linked Antibody | CST | 7076 | 1:2000 | 1:1000 |

**Supplementary table 2. Primer sequence for target gene.**

| Target | Forward primer (5′-3′) | Reverse primer (5′-3′) |
| --- | --- | --- |
| U6 | CCTTCGGGGACATCCGATAAAA | TCGATTTGTGCGTGTCATCC |
| GAPDH | TTGCCCTCAACGACCACTTT | TGGTCCAGGGGTCTTACTCC |
| miR-424-5p | AGGGGATACAGCAGCAATTCA | ACCTTCTACCTTCCCCACGA |
| SOCS5 | TCAACTCCTCAGCAACAA | GGTAACACAAGAATCATTATCCT |
| SOCS6 | GCTCGGCTTCTCATAATG | GAGGCATAAGTCCAATAACG |
| TP53 | AGTATTTGGATGACAGAA | ATGTAGTTGTAGTGGATG |

**Supplementary table 3. Information on the binding of *TP53* to miR-424-5p host gene.**

| Name | Score | Relative Score | Stat | End | Strand | Predicted sequence |
| --- | --- | --- | --- | --- | --- | --- |
| MA0106.2.TP53 | 9.688 | 0.832 | 1081 | 1095 | - | TCATGTCCTGAAATG |
|  | 7.959 | 0.811 | 841 | 855 | + | ACTTGTGCTGAAATG |
