# Supplementary figures and images for: TP53 mitigates cisplatin resistance in non-small cell lung cancer by mediating the effects of resistant cell-derived exosome mir-424-5p
Source: Heliyon. 2024 Feb 22;10(5):e26853. doi: 10.1016/j.heliyon.2024.e26853 (PMC10909722; doi:10.1016/j.heliyon.2024.e26853)

Figure 3D

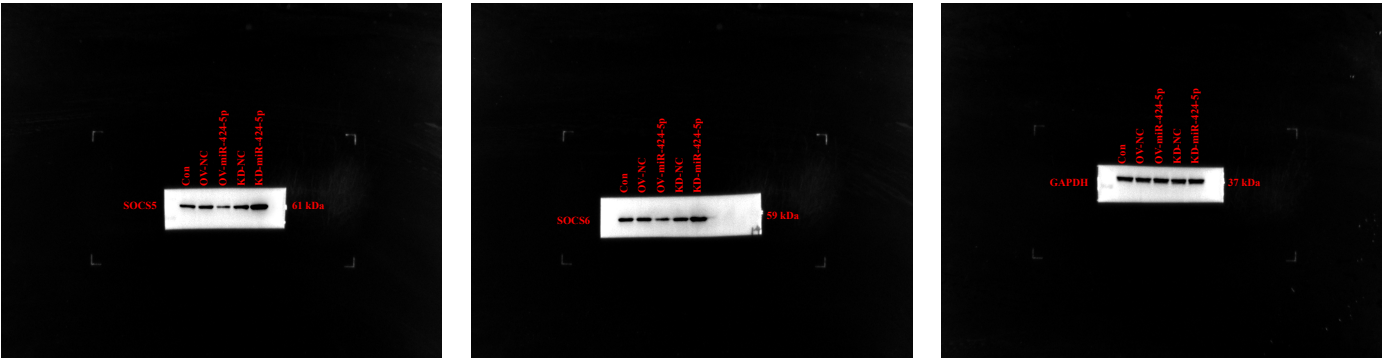

Figure 4A

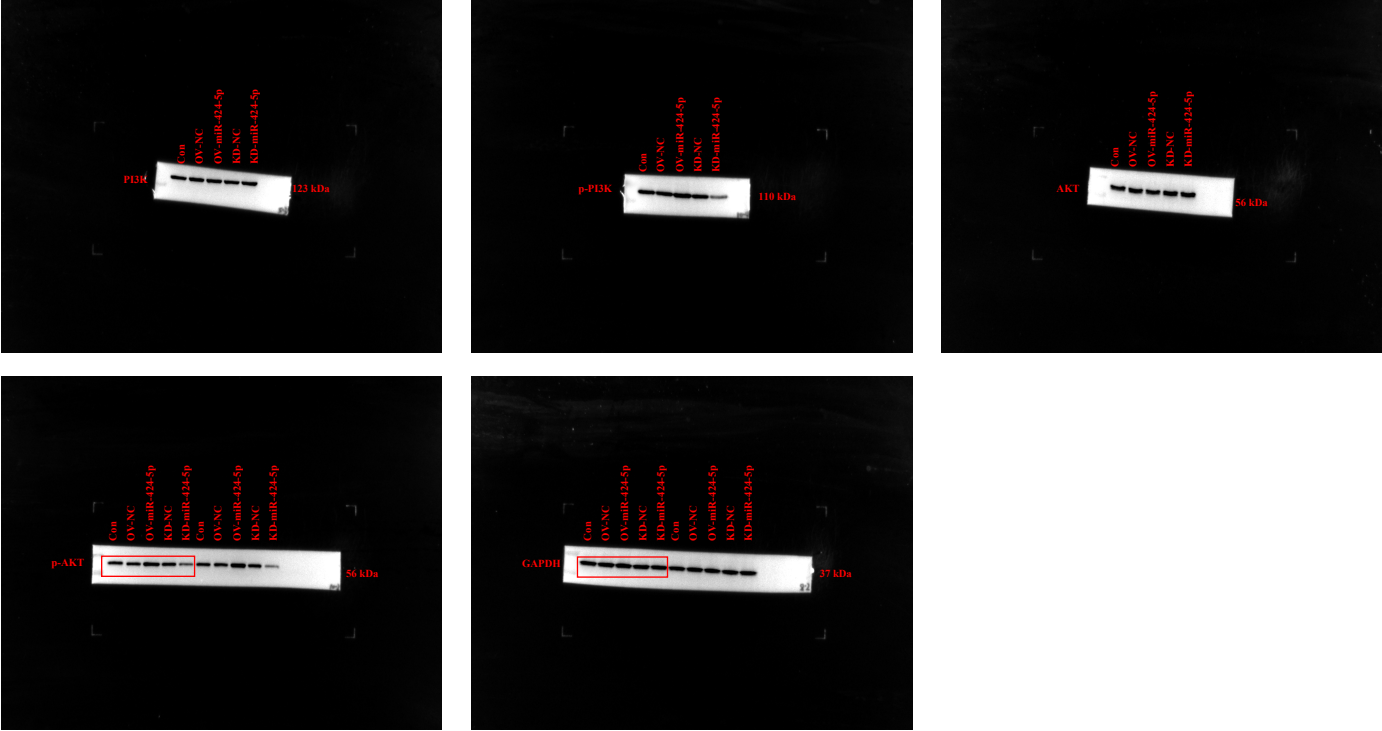

Figure 4B

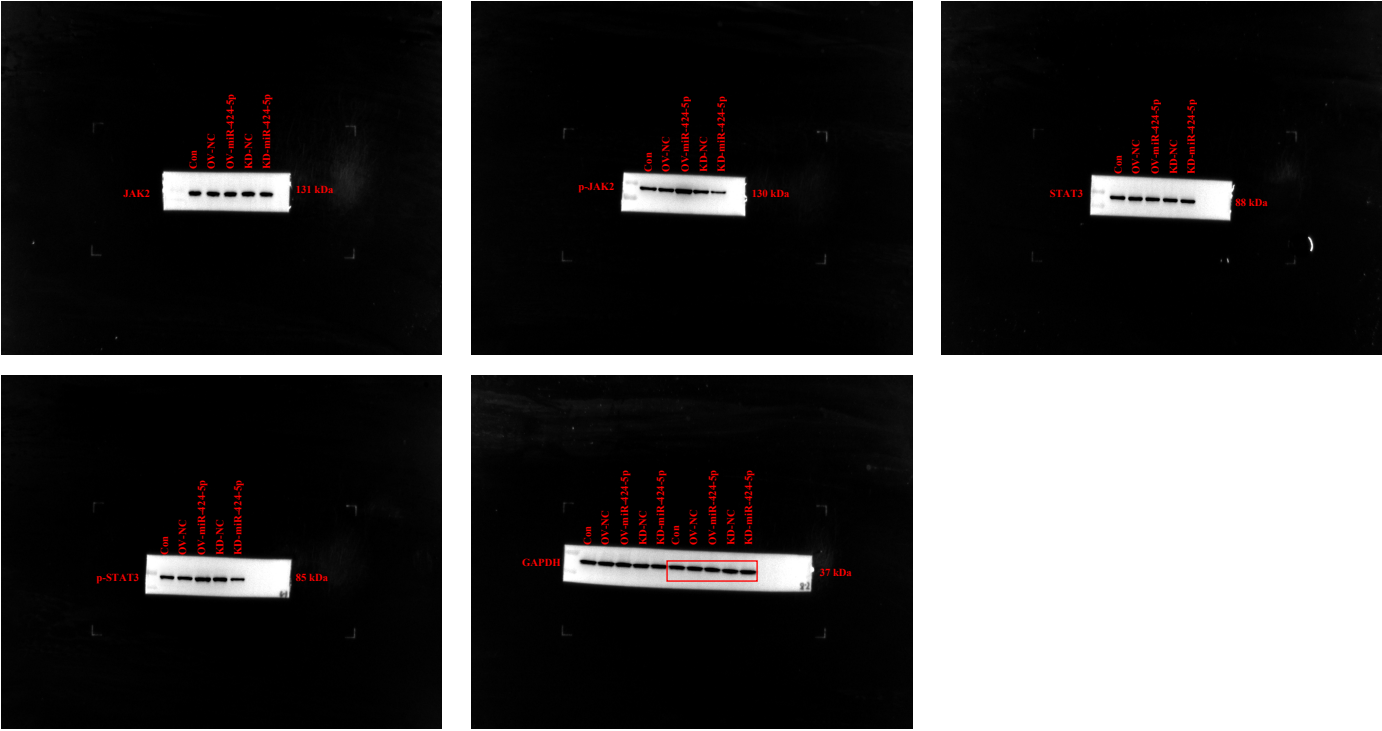

Figure 5E

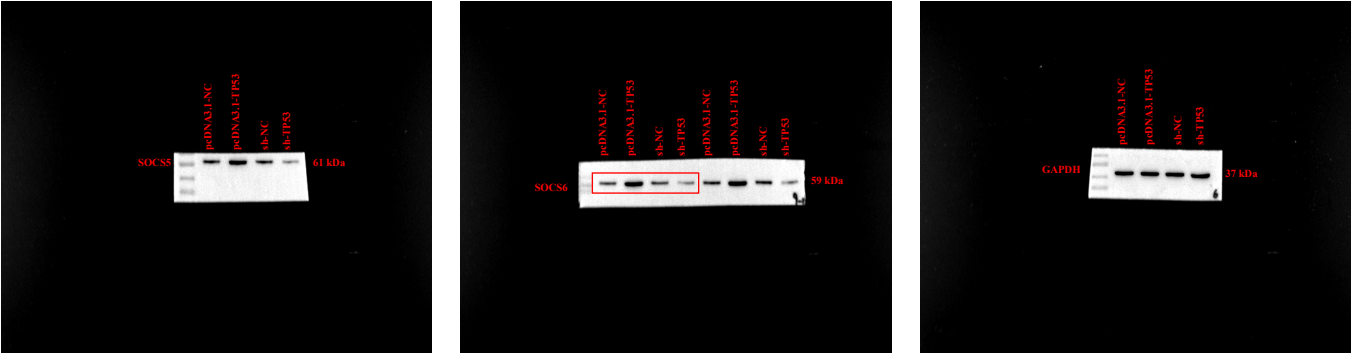

Figure 5F

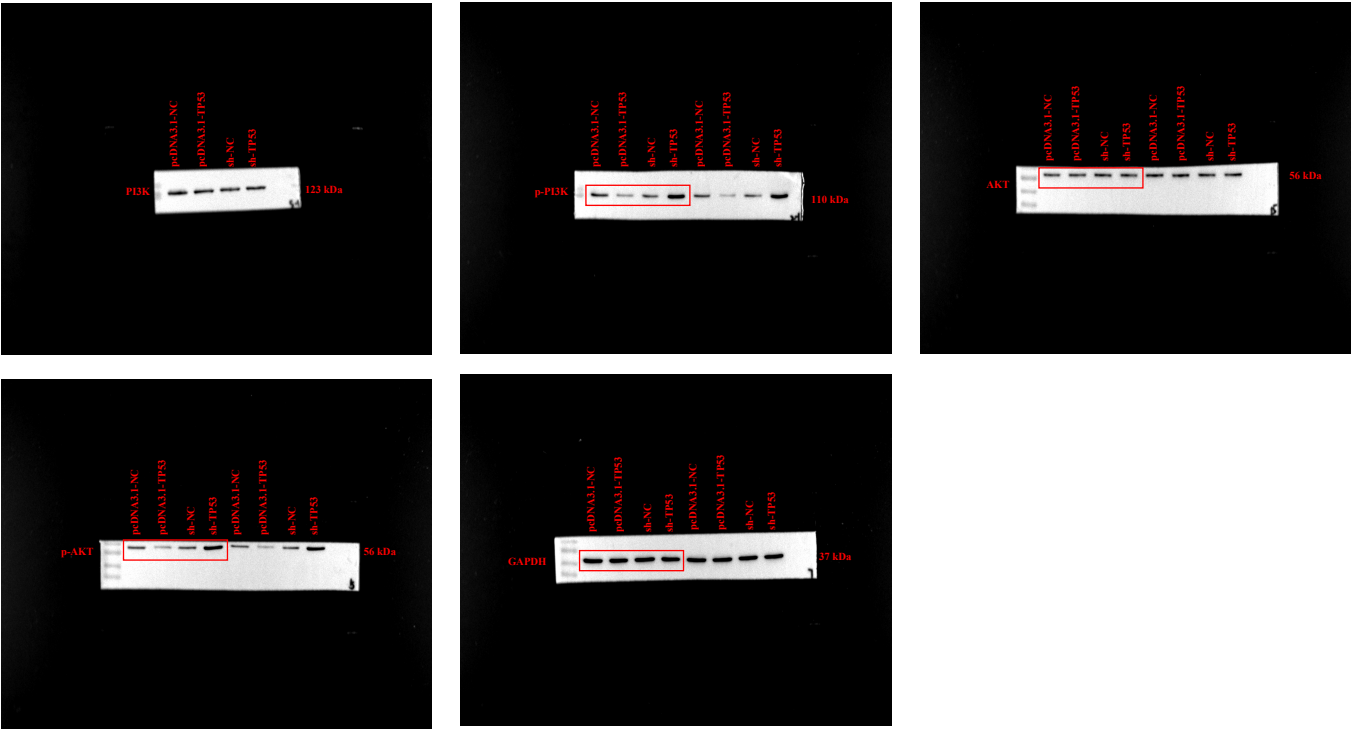

Figure 5G

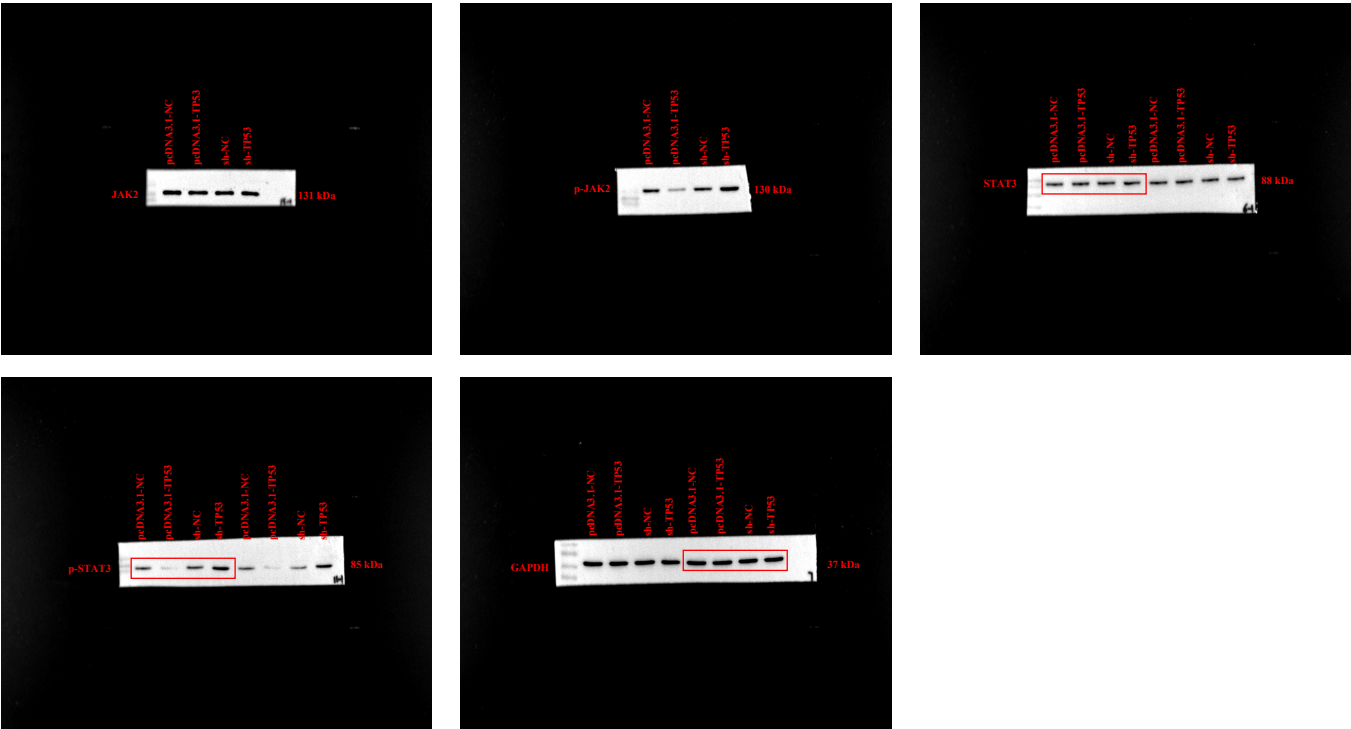

Figure 7B

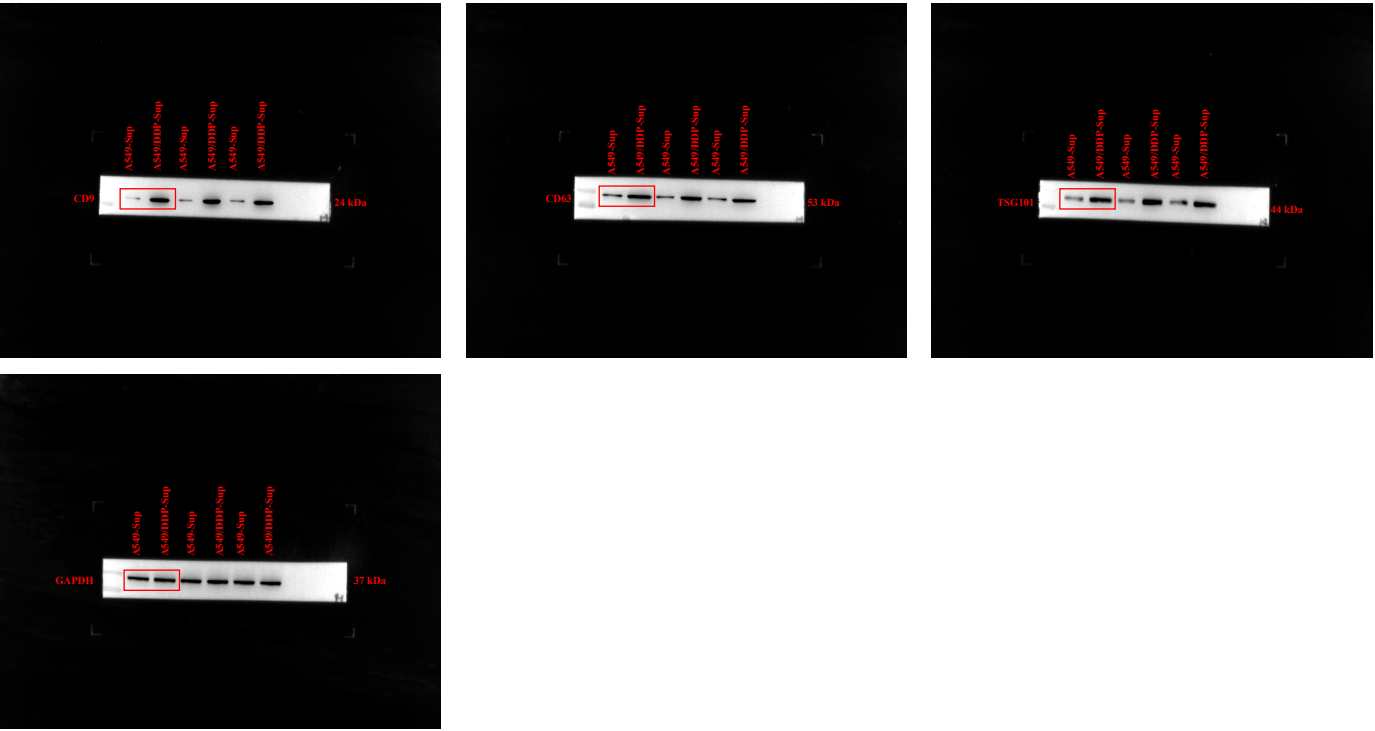

Supplement: Multimedia component 2 [file mmc2.pdf]
